# The Impact of Excessive Fructose Intake on Adipose Tissue and the Development of Childhood Obesity

**DOI:** 10.3390/nu16070939

**Published:** 2024-03-25

**Authors:** Anna Karenina Azevedo-Martins, Matheus Pedro Santos, Julie Abayomi, Natália Juliana Ramos Ferreira, Fabiana S. Evangelista

**Affiliations:** 1 Group of Study in Endocrinology and Metabolism, School of Arts, Sciences and Humanities, University of São Paulo, São Paulo 03828-000, Brazil; rivermathe@gmail.com (M.P.S.); rms.natalia@gmail.com (N.J.R.F.); fabiana_evangelista@yahoo.com.br (F.S.E.); 2 School of Medicine and Nutrition, Faculty of Health, Social Care and Medicine, Edge Hill University, Ormskirk L39 4QP, UK; abayomij@edgehill.ac.uk

**Keywords:** fructose, childhood obesity, adipose tissue

## Abstract

Worldwide, childhood obesity cases continue to rise, and its prevalence is known to increase the risk of non-communicable diseases typically found in adults, such as cardiovascular disease and type 2 diabetes mellitus. Thus, comprehending its multiple causes to build healthier approaches and revert this scenario is urgent. Obesity development is strongly associated with high fructose intake since the excessive consumption of this highly lipogenic sugar leads to white fat accumulation and causes white adipose tissue (WAT) inflammation, oxidative stress, and dysregulated adipokine release. Unfortunately, the global consumption of fructose has increased dramatically in recent years, which is associated with the fact that fructose is not always evident to consumers, as it is commonly added as a sweetener in food and sugar-sweetened beverages (SSB). Therefore, here, we discuss the impact of excessive fructose intake on adipose tissue biology, its contribution to childhood obesity, and current strategies for reducing high fructose and/or free sugar intake. To achieve such reductions, we conclude that it is important that the population has access to reliable information about food ingredients via food labels. Consumers also need scientific education to understand potential health risks to themselves and their children.

## 1. Introduction

According to the World Obesity Federation [1], childhood obesity is predicted to rise globally, especially among male children and adolescents (aged 5–19 years), from 103 million in 2020 to 208 million in 2035 and from 72 to 175 million in female children and adolescents, thus representing a major worldwide public health concern. Childhood obesity diagnosis is based on body mass index (BMI) percentile considering children’s age and sex [2], and its occurrence is associated with non-communicable health complications such as cardiovascular disease, type 2 diabetes mellitus (T2DM), mental health problems, and some types of cancer in later life [3,4]. These conditions are usually observed in adults, but the development of non-communicable disease (NCD) often begins in childhood. In children, excessive energy intake and/or a sedentary lifestyle can lead to positive energy balance with consequent expansion of adipose tissue beyond what is expected in a healthy child [5]. 

Since treating this complex multifactorial condition [6,7] is so challenging, preventing childhood obesity is fundamental for improving public health worldwide. It is also important to successfully control obesity escalation since a child living with obesity tends to become an adult living with obesity [3,4]. Although the causes of obesity are varied, the increasing consumption of fructose [8] has become a pivotal issue in understanding the pandemic status of obesity [6,9,10] and the exponential increase in childhood obesity [11]. Indeed, experimental protocols, epidemiological studies, and clinical trials have provided convincing evidence that sugar-sweetened beverages (SSBs) increase the risk for obesity [12]. Here, we discuss the impact of excessive fructose intake on the biology of adipose tissue and its contribution to the development of childhood obesity.

## 2. Biology of Adipose Tissue

Adipose tissue can be classified into white adipose tissue (WAT) and brown adipose tissue (BAT). WAT is distributed throughout the body in distinct depots, including visceral (vWAT) and subcutaneous WAT (sWAT) depots. vWAT is in omental, mesenteric, retroperitoneal, gonadal, and pericardial regions, while sWAT is subcutaneous and, in humans, also in the abdomen and gluteofemoral regions [13,14]. BAT is typically found in the interscapular and supraclavicular regions and represents approximately 4.3% of the fat mass in humans [15].

White adipocytes are responsible for the storage and release of lipids in response to systemic nutritional and metabolic needs, and brown adipocytes are specialized in thermogenesis. The brown adipocyte’s function is due to the expression of the uncoupling protein 1 (UCP1), which decouples the proton gradient from the mitochondrial ATP synthesis, dissipating energy in the form of heat [15]. A third type of adipocytes has characteristics of both brown and white adipocytes (known as “beige” or “brite” adipocytes) and can be found in WAT depots. In addition, the morphological and functional characteristics of these beige adipocytes are very similar to those of brown adipocytes, including thermogenic capacity due to the expression of UCP1 [16].

In addition to the function of acting as an energy reservoir for protecting vital organs and controlling body temperature, adipose tissue is also an endocrine organ that produces and secretes many signaling proteins called adipokines. These proteins modulate various functions in the body, including systemic metabolic state, inflammatory response, and cardiovascular function [17]. The role of adipokines such as leptin, adiponectin, interleukin-6 (IL-6), and fibroblast growth factor 21 (FGF21) in health and disease were widely presented in a recent review by Clemente-Suárez et al. [17]. 

The development and expansion of adipose tissue can be driven by the formation of new adipocytes from preadipocyte differentiation in the process of adipogenesis (hyperplasia) and by increasing lipid storage via lipogenesis (hypertrophy). On the other hand, the process of lipolysis, which is the hydrolysis of triacylglycerol followed by fatty acid release to generate energy, is involved in the reduction in adipocyte size. Fat accumulation is determined by the balance between the synthesis and breakdown of triacylglycerol [18].

Fetal adipogenesis begins early in the second trimester of pregnancy in a cranial to caudal and then medial to lateral manner [19]. In the 28th week of pregnancy, WAT is present in the principal fat depots throughout the body [19], while BAT can be identified earlier in development [20]. Considering that adipogenesis involves different precursors and distinct regulatory processes, factors such as maternal nutrition state and obesity can influence the developmental patterning of fetal adipose tissue with future consequences for adulthood [21].

Two weeks after birth, the expansion of adipose tissue rises rapidly in response to increased nutrient availability in the postnatal environment [22]. Adipocyte proliferation in human adipose tissue is markedly observed before two years and during puberty [23,24]. After puberty, adipocyte number and size become relatively static in lean individuals [25]. However, increases in adipose mass in individuals living with obesity can result from both adipocyte size and number elevation [26]. 

Regarding lipogenesis, fatty acids and monoacylglycerols taken up from the circulation are driven toward triacylglycerol synthesis and storage in the adipocyte. In this process, fatty acids are acylated with CoA, forming acyl-CoA via acyl-CoA synthetase (ACS) [27]. Then, the enzymes glycerol-3 phosphate acyltransferase (GPAT) and diacylglycerol acyltransferase (DGAT) are responsible for the final part of the process, esterifying acyl-CoA together with glycerol-3-phosphate in triacylglycerol [28]. 

Carbohydrates can be converted to fatty acids via a process known as de novo lipogenesis. This process occurs via the production of citrate followed by its conversion into acetyl-CoA by the enzyme ATP citrate lyase (ACL) [29]. Via the action of the enzyme fatty acid carboxylase (ACC), the acetyl-CoA formed is converted to malonyl-CoA, which will be used as a substrate for the synthesis of triacylglycerol by the enzyme fatty acid synthase (FAS) [28].

At the early stage of human pregnancy, the amount of fetal fat is provided by the transplacental transfer of fatty acids. However, during the last 3 months, fetal fatty acids are derived predominantly from their own synthesis de novo [30]. In addition, the glucose used in lipogenesis is delivered by the mother via the placenta because the fetus does not synthesize glucose. Thus, conditions such as maternal obesity or gestational diabetes can impair glucose metabolism in fetal adipose tissue and consequently increase adiposity and result in elevated birth weight [31].

The regulation of lipolysis has been extensively reviewed in another previous study [32]. Briefly, lipolysis starts from the binding of noradrenaline to beta-adrenergic receptors, which allows for the increase in cyclic AMP and the activation of protein kinase A (PKA). PKA phosphorylates hormone-sensitive lipase (HSL) and perilipins, thus allowing triacylglycerol hydrolysis into fatty acids. Adipocyte triacylglycerol lipase (ATGL) and monoacylglycerol lipase (MAGL) also play important roles in lipolysis, with ATGL participating in the initial phase of triacylglycerol hydrolysis by cleaving the first fatty acid and MAGL cleaving the last fatty acid of the triacylglycerol [32]. The three liberated fatty acids may be oxidized in muscle or BAT, and the glycerol may be used as a precursor for gluconeogenesis in the liver [33].

Lipolysis occurs during periods of energy demand, such as fasting and physical exercise, but seems to be suppressed during fetal life. In fact, adipose tissue favors lipid synthesis over lipolysis under normal conditions, actively contributing to progressive fat storage during the last weeks of gestation [34]. While in adults catecholamines are the main lipolytic hormones, thyrotropin hormone plays a key role in lipolysis in newborns. However, during childhood, catecholamines increase and replace their effect [35].

## 3. Biochemical Aspects of Fructose

Fructose is a monosaccharide made up of six carbon atoms bonded by single covalent bonds, presenting hydroxyl groups and a carbonyl group formed by a double bond between carbon and oxygen. The position of this grouping will determine, after hydrolysis of the monosaccharide, if it will give rise to ketone or aldehyde. Fructose, also called ketohexose, when hydrolyzed, gives ketone, since it contains the carbonyl group at the end of the chain. Glucose, on the other hand, when hydrolyzed, will give rise to aldehyde, and is called aldohexose. In the same way as glucose, 1 g of fructose provides 16 kJ of energy [36].

Usually, fructose is ingested as a monosaccharide or disaccharide (combined with glucose) via sucrose [37]. It can also be found in tri- and tetrasaccharides, such as raffinose and stachyose, and is present in legumes, soybeans, lentils, peas, and beans, for example [38]. When sucrose is the source of fructose, it must be digested to liberate fructose and glucose for absorption. Dietary fructose is absorbed and transported via facilitative glucose transporters (GLUTs) located on both sides of the enterocyte membrane [39]. The absorption of fructose is performed by GLUT5, located on the enterocyte’s apical membrane, and transported to the portal bloodstream by GLUT2 present on the basolateral membrane of these cells [40]. GLUT5 exclusively transports fructose, while GLUT2 transports both glucose and fructose, as well as galactose. When co-ingested with glucose, intestinal fructose absorption is increased due to the upregulation of GLUT5 [41]. Unlike glucose, the uptake of fructose into enterocytes is an insulin- and sodium-independent process without the expenditure of ATP [42].

**Figure 1:**
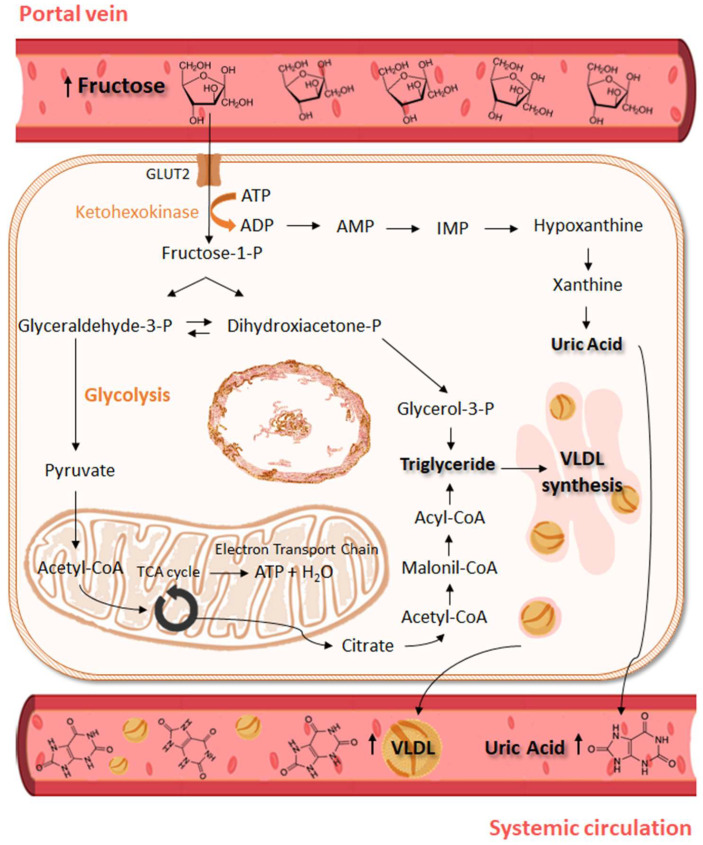
Consequences of high fructose intake for hepatic metabolism. After meals, the fructose absorbed and not metabolized in the intestine is directed to the liver via the portal vein and enters the hepatocyte via GLUT2, to then be phosphorylated to fructose-1-P by ketohexokinase. High fructose phosphorylation rates may cause ATP depletion, leading to purine degradation, uric acid formation, and its release to the systemic circulation. Cleavage of fructose-1-P forms dihydroxyacetone-P and glyceraldehyde that are then converted to glyceraldehyde-3-P, which participates in the glycolytic process to form pyruvate. In mitochondria, pyruvate is converted to acetyl-CoA that enters the tricarboxylic acid cycle (TCA cycle), resulting in ATP generation in the mitochondrial electron transport chain. However, after intense fructose oxidation, the citrate produced in the mitochondria can be displaced into fatty acid synthesis. Citrate exits mitochondria and is reconverted to acetyl-CoA. In the cytoplasm, acetyl-CoA is converted to malonyl-CoA, which ultimately forms triglyceride and VLDL (very low-density lipoprotein) in the endoplasmic reticulum. VLDL is exported to systemic circulation. Triglyceride esterification is accentuated by dihydroxyacetone-P conversion to glycerol-3-P. Arrows indicate the flow of chemical reactions.

Most of the fructose absorbed by enterocytes is directed to the liver, where it participates in carbohydrate metabolism and de novo lipogenesis. Hepatic fructose is rapidly phosphorylated by ketohexokinase (KHK) to fructose-1-phosphate (F1P), which is further metabolized to glyceraldehyde 3-phosphate and dihydroxyacetone phosphate by aldolase-B. Later, these products are used in glycolytic pathways or even in lipogenic processes, especially during high fructose intake (Figure 1) [43]. The remaining fructose in the enterocyte is also metabolized by KHK to F1P and processed to further metabolites such as short-chain fatty acids (SCFAs), glucose, and organic acids via aldolase-B and triokinase sequentially [44]. 

A recent study by Jang et al. [45] showed that with low-dose fructose intake (<0.5 g/kg), the intestine can metabolize about 90% of this nutrient, mainly into glucose and organic acids, releasing only a nonmetabolized small part into the portal circulation. Conversely, with a high intake of fructose (≥1 g/kg), the intestinal capacity to metabolize this nutrient is exceeded, releasing the excess fructose to the liver [45]. Another problem with ingesting large amounts of fructose is that it is likely to exceed the absorptive capacity of the intestines, which is thought to be about 5–50 g per serving in a healthy adult [46]. The incomplete absorption of fructose is associated with diarrhea [47] and other gastrointestinal symptoms, such as gas accumulation, flatulence, and abdominal pain [48].

## 4. Sources and Consumption of Fructose

The main sources of fructose in the diet are fruit juice, fruit, yogurt, honey, ice cream, confectionery, and soft drinks sweetened with either sucrose or high-fructose corn syrup (HFCS) [49]. Naturally occurring fructose found in yogurt and fruit was shown to be protective against cardiometabolic disease, so there is no advice to limit these foods in the diet [49]. However, in recent decades, the dietary consumption of sugar has increased, and this is often blamed for the increased prevalence of obesity and cardiometabolic disease [50]. The consumption of fructose in children and adolescents aged 2–18 years exceeds the desirable goal of less than 5% energy intake from free sugars [51]. Therefore, healthier approaches to beverage and dietary consumption should be adopted in infancy [51] to avoid the development of obesity and comorbidities [11].

Recommendations for reducing sugar intake focus on “free sugar” rather than the intrinsic sugars found in milk and plant food. Both the World Health Organization [52] and the UK’s Scientific Advisory Committee on Nutrition [53] recommend limiting free sugar intake to less than 5% of overall energy intake. Free sugar is defined as sugar added by the manufacturer, cook, or consumer, plus sugar found in honey, syrup, or fruit juice [49,54]. It is often added as a sweetener to food and drink in the form of sucrose (table sugar) or HFCS (55% fructose), which are commonly used in sugar-sweetened beverages (SSBs) [37]. It is estimated that Americans consume 25 kg of HFCS per person per year [55]. Most European countries consume far less HFCS, such as the United Kingdom (UK), consuming <0.5 kg per person per year [55]. However, nutritional surveys in the UK show that most people consume far more free sugar compared to the recommendations, with a mean intake of 12.5% of energy coming from free sugar, including 9% and 3.5% of energy from sucrose and fructose, respectively [54]. There appear to be no figures for fructose consumption in Brazil, but the consumption of SSBs is reported to be high. In fact, young Brazilians consume an average of 281.5 mL or 1.6 SSBs per day, which contributes to 5.9% of energy intake [56].

## 5. Excessive Fructose Intake and Its Metabolic Implications

**Figure 2:**
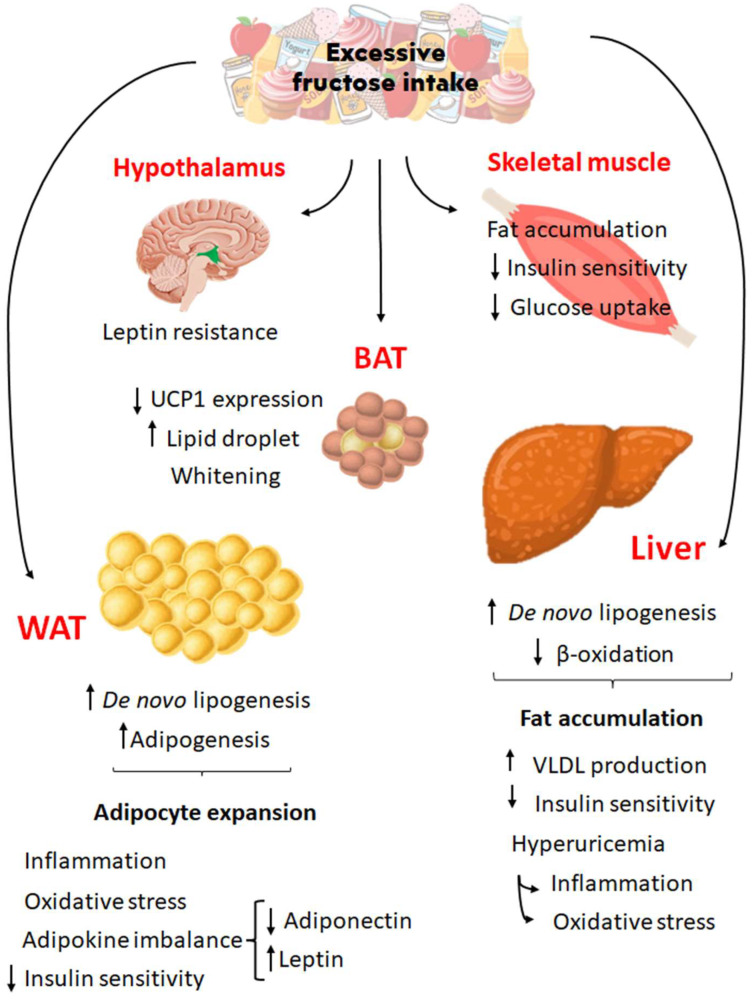
Effects of excessive fructose intake on different organs and tissues. High fructose intake affects the metabolic process via several pathways in organs such as the hypothalamus, skeletal muscle, liver, BAT, and WAT, contributing to an obesity phenotype and metabolic syndrome development. BAT, brown adipose tissue; WAT, white adipose tissue; UCP1, uncoupling protein 1; VLDL, very-low-density lipoprotein. Up arrows indicate increased levels of the respective process, while down arrows indicate decreased levels of the respective process.

Excessive fructose intake has been associated with several diseases [8,37], such as metabolic syndrome [57], blood hypertension [58], hypertriglyceridemia [59], non-alcoholic fatty liver disease (NAFLD) [60], and obesity in adults and infants [12] (Figure 2). 

Most of the fructose that exceeds the intestinal ability to absorb and metabolize this nutrient will be driven to the liver, stimulating pathways that will simultaneously lead to hepatic fat accumulation and reduction in hepatic fat removal [60]. Fructose stimulates sterol-regulatory element-binding protein 1 (SREBP-1c) and carbohydrate-responsive element–binding protein (ChREBP), both of which are key transcriptional regulators of hepatic de novo lipogenesis [43]. On the other hand, fructose upregulates the synthesis of acetyl-CoA, which is then converted to malonyl-CoA, leading to β-oxidation inhibition via limiting carnitine palmitoyl transferase action [61]. VLDL and triglyceride (TG) production are accentuated by the formation of glyceraldehyde-3-phosphate, a substrate for its synthesis from fructose-1-phosphate [61] (Figure 1).

In healthy subjects, a high-fructose diet consumed for 7 days resulted in an increase in ectopic fat accumulation in liver and skeletal muscle, increased fasting plasma TG and VLDL, and a decrease in hepatic insulin sensitivity, independently of family history of T2DM [62]. Glucose metabolism and uptake pathways may be impaired by dyslipidemia status generated from excessive fructose intake [63]. Mice fed a 66% fructose diet for 2 weeks decreased the number of insulin receptors in skeletal muscle and liver, as well as increased blood pressure and TG serum levels [64]. In addition, 28 days of high-fructose feeding reduced the insulin-stimulated phosphorylation of insulin receptor substrate (IRS 1/2) in the skeletal muscle and liver of mice. Those mechanisms might be involved in fructose contributing to insulin resistance and T2DM pathogenesis [65].

Fructose metabolism in the liver forms uric acid (UA) as a metabolite (Figure 1), which consequently induces endothelial dysfunction, inflammation, and oxidative stress in hepatocytes [66]. By activating the proinflammatory NF-κB signaling cascade, hyperuricemia increases the expression of inflammatory biomarkers such as C-reactive protein, fibrinogen, ferritin, and complement C3 in HepG2 cells [67], which could contribute to the inflammation observed in metabolic and cardiovascular diseases. In accordance, fructose administration for 20 weeks in rats induced hyperuricemia, increased serum proinflammatory cytokines IL-6, TNF-*α*, and MIP-2, and decreased anti-inflammatory cytokine IL-10 [68].

## 6. Fructose and Obesity: What Happens with Adipose Tissue

High fructose consumption and obesity development in modern societies have become indissociable [69]. This highly lipogenic sugar stimulates adipogenesis and results in WAT accumulation [70] and may also cause WAT inflammation [71], oxidative stress [69], dysregulated adipokine release [72], and BAT whitening [73], involving different tissues and metabolic pathways (Figure 2). 

In mice, a 15% increase in fructose intake via water consumption increased adiposity [70], while a high-fat (31% g/kg) and high-fructose (24% g/kg) diet increased WAT accumulation, and adipocyte hypertrophy, glucose intolerance and reduced insulin secretion by isolated pancreatic islets [74]. Moreover, rats fed a fructose-rich diet for 8 weeks showed increased epididymal and mesenteric WAT, with more large adipocytes and fewer small adipocytes in visceral WAT, compared to controls [75]. Increased adipocyte size following exposure to a fructose-rich diet was also observed in rat retroperitoneal WAT, with an increased number of adipocyte precursor cells (APCs) and adipogenic potential, inferred by the higher expression level of two adipogenic competency markers, PPAR*γ*2 and Zfp423 [76]. Additional data are necessary to elucidate how different visceral or subcutaneous WAT deposits respond to high fructose intake.

Fructose-stimulated adipogenesis is also mediated by the increasing of active glucocorticoid (GC) levels via 11β-hydroxysteroid dehydrogenase type 1 (11β-HSD1) activity in WAT [77]. Fructose metabolism in 3T3-L1 adipocytes [78] and epidydimal WAT [79] generates NADPH that is required for the activation of 11β-HSD1, an enzyme that converts inert GC metabolite into active hormone, resulting in high levels of plasma GC [77]. GCs are required to fully differentiate adipocytes by induction of key adipogenic transcription factors such as C/EBPα, C/EBPβ, C/EBPδ, KLF5, KLF9, and PPARγ in the early phase of differentiation [80,81]. In vitro, 3T3-L1 preadipocytes cultured with only fructose as the carbohydrate source differentiated into adipocytes [82].

High-fat and high-fructose diets have a negative impact on the metabolome of the liver, muscle, BAT, and WAT [83], and long-term fructose consumption increased mRNA levels of genes involved in fatty acids synthesis such as FAS and SCD1 and decreased the expression of genes involved in lipid mobilization like ATGL and HSL, thereby contributing to adipocyte enlargement [84]. A high-fructose diet for 3 weeks increased adipocyte area, FAS activity, and GLUT5 expression in rats [85]. Large adipocytes and increased plasma TG persisted even after fructose withdrawal from the diet, which might indicate a longstanding negative effect of a fructose-rich diet in the young that may persist into adulthood [85].

Excessive fructose intake also might contribute to low-grade systemic inflammation. Male and female rats fed a high-fructose diet for 24 weeks presented a visceral WAT accumulation with high concentrations of inflammatory markers such as iNOS, TNFα, IL-1β, IL-18, MDA, and ALT [71]. Kovacevi et al. [86] found that visceral WAT inflammation precedes obesity in female rats maintained with 10% added fructose in water. In this study, inflammatory markers like IL-1β, IL-6, TNFα, and the nuclear accumulation of NFκB were higher even before increases in visceral adiposity. The authors also observed insulin resistance in visceral WAT, detected by IRS1 inhibitory phosphorylation and decreased Akt activity, and hyperuricemia [86], which in turn activates the NFκB inflammatory pathway [67], and may be a key factor responsible for the proinflammatory endocrine imbalance in WAT [87]. Local inflammation was found in WAT from high-fructose-fed rats, with increased TNF-α levels, MPO activity, and decreased adiponectin, an anti-inflammatory molecule, that persisted after fructose diet withdrawal [85]. 

Singh et al. [88] found that a 60% fructose diet, fed over 10 weeks, induced the already-mentioned fructose effects (visceral adiposity, insulin resistance, and increased TG levels) accompanied by the activation of NLRP3 in rat epidydimal adipose tissue. In turn, NLRP3 inhibition mitigated inflammatory signaling and lipogenesis in WAT, as well as improved insulin sensitivity [88]. NLRP3 is an intracellular sensor that, when activated, forms an inflammasome complex and results in caspase-1 expression and the secretion of IL-1β and IL-18 in macrophages [88]. Therefore, NLRP3 represents another mediator of high-fructose deleterious effects, including the increased lipogenesis rates that contribute to the development of obesity. However, the relationship between high fructose ingestion and inflammation in humans is less clear. The excessive consumption of fructose over a period of 8 days from SSBs did not worsen the low-grade chronic systemic inflammation or increase body weight in adults, probably due to the short period of nutrient ingestion [89].

In obesity, systemic oxidative stress is accentuated by several mechanisms, including the activation of NADPH oxidase (NOX) with superoxide production as a consequence of elevated FFA, inflammatory cytokines, or hyperglycemia [90]. Fructose-induced obesity caused oxidative stress in hypertrophic visceral adipose tissue in male Wistar rats [91], and ROS production in cultured macrophages was higher in fructose-treated cells compared with untreated or glucose-treated cells [92]. Hyperuricemia might mediate this fructose induction of oxidative stress [69] since UA is involved with ROS production by activating NOX and reducing endothelial levels of the NO [93].

Additionally, fructose increases the production of GC [77], such as dexamethasone and cortisol, which also play a role in inducing the overproduction of ROS [94]. Such oxidative stress status could be implicated in adipogenesis and lipid accumulation in adipocytes, representing another link between high fructose intake and obesity development [69]. In addition, oxidative stress in 3T3-L1 cells mediated the expression of pro-adipogenic genes PPARγ, RXRα, and C/EBPα and resulted in adipogenesis and lipid accumulation [95], and the impairment of ROS production decreased the expression of adipogenic markers and lipid deposition in this cell line [96]. 

The profile of adipokines produced by different adipose tissue depots reflects their health status [97]. WAT from lean subjects releases an anti-inflammatory adipokine profile with high levels of adiponectin, IL-10, and IL-4 and low levels of leptin, TNF-α, IL-6, and MCP-1, while the WAT from obese releases the same adipokines but in inverse proportion [98]. Interestingly, high-fructose intake has also been associated with damaged adipokine profile secretion [72]. Fructose may lead to reduced adiponectin levels [85] as a consequence of hyperuricemia [87], impairing its anti-inflammatory and antidiabetic effects [99]. Adiponectin is very sensitive to fructose intake since a single high-fructose meal in rats was enough to reduce its serum concentration after 2 or 4 h, as well as increasing TNF-α content and neutrophil recruitment in the liver [100]. These effects limit the potential of adiponectin to decrease hepatic gluconeogenesis and WAT inflammation or increase fatty acid oxidation in skeletal muscle and liver, contributing to the maintenance of obesity phenotype [101].

WAT from individuals with obesity increases leptin secretion, leading to leptin resistance by desensitizing its receptors in the hypothalamus, prejudicing its regulation of food intake and contributing to obesity [102]. High fructose intake also seems to induce hyperleptinemia in rats [72,103,104] by leptin gene overexpression in WAT, which was further decreased by treatment with hypouricemic agents, suggesting that fructose-induced hyperuricemia is directly associated with hyperleptinemia [103]. Since this effect was not observed in other studies, further investigations are required [105,106]. Despite that, high fructose ingestion induced peripheral leptin resistance in rats, in combination or not with high-fat diets [104,105,106,107]. This effect was reversed after fructose withdrawal from the diet [104]. Intraperitoneal leptin injections successfully reduced food intake in adult rats fed a fructose-free diet but had no effect in animals fed a 60% fructose diet for 6 months. This leptin resistance was associated with decreased phosphorylation in the hypothalamic signal transducer and activator of transcription 3 (STAT3) [105]. When fructose was added into the diet of post-weaning rats, it resulted in decreased leptin receptors and SOCS3 in WAT, suggesting that a long-term fructose diet alters paracrine signaling of leptin [106], alerting to the risks of high fructose consumption already early in life.

Recent studies indicate an effect of fructose also in BAT metabolism [73,108,109,110]. C57BL/6 mice fed a high-fructose diet (50% of energy as fructose) for 12 weeks showed an interscapular BAT (iBAT) whitening process, with a reduction in UCP1 immunodensity [73]. A similar study from the same group did not find a pronounced whitening process in high-fructose-fed mice, only in high-fat-fed animals. However, a high-fructose diet caused lipid accumulation in iBAT and had negative immunostaining for vascular endothelial growth factor A (VEGF-A), which could indicate a hypoxic state that precedes mitochondrial loss and suggests that whitening might occur in prolonged high fructose intake. Those effects were reduced after PPAR-α activation, known to induce mitochondrial biogenesis, β-oxidation, fatty acid uptake, and UCP1 expression [108].

Additionally, in high-fructose-fed mice, a proportional reduction in BAT mass was observed in comparison with visceral WAT and morphological remodeling, with increased lipid deposition in enlarged intracellular lipid droplets [109]. In humans, a 14-day high-fructose diet impaired glucose uptake in BAT without changes in cold-stimulated thermogenesis [110]. Yet, other studies are required to confirm long-term high fructose intake effects on BAT metabolism, especially in humans, from childhood to adulthood.

## 7. The Multiple Causes of Childhood Obesity

Obesity is a multifactorial condition; therefore, some factors such as maternal lifestyle during the pre-gestational and gestational phases, birth weight, nutrition and physical activity, and socioeconomic and genetic factors are associated with childhood obesity pathogenesis [111,112].

Prenatal and early postnatal factors increase the risk of developing obesity and several additional diseases throughout life [111,113]. Children born from mothers with obesity or from women who developed gestational diabetes are more likely to develop obesity and metabolic problems compared to children born from healthy mothers [113]. Therefore, factors such as pre-pregnancy BMI, maternal weight gain, and glucose metabolism during pregnancy and lactation correlate with the occurrence of childhood obesity [114,115,116]. Additionally, there is a greater propensity for obesity in adulthood in children with high birthweight and an increase in the central distribution of fat in those with low birthweight, reaffirming that the gestational phase is an important moment for an increase in later body adiposity [113]. 

Birthweight is an indicator of fetal health that reflects both intrauterine growth and gestational age. Abnormal fetal growth is correlated with an increased risk for cardiometabolic disease [112,113], and there is a linear association between birth weight and BMI in adulthood [117].

Since a lack of physical activity and an inadequate diet are closely related to energy balance, these are often identified as the main risk factors for obesity in childhood [112]. Diets that lack a good fruit and vegetable intake, alongside increased consumption of ultra-processed foods and SSBs, are thought to be among the various nutritional patterns that contribute to childhood obesity [118]. Since the family is primarily responsible for the child’s development and the provision of food and children look to their parents for examples, parental involvement in lifestyle changes and obesity prevention is essential. Promoting an environment that prioritizes a balanced diet and encourages the practice of regular physical activity are essential ingredients for obesity prevention [119].

Low socioeconomic status is also identified as one of the risk factors for childhood obesity since individuals from lower socioeconomic groups tend to have a different lifestyle than individuals with higher purchasing power. Individuals in higher socioeconomic groups can have improved access to more nutritional diets that contribute to better food choices and greater opportunities for regular physical activity, thus reducing the risk of overweightness and obesity [112,120].

In addition to environmental factors, genetic factors are involved with excessive weight gains, such as the mutations in the genes for leptin (LEP) and leptin receptors (LEPR), proopiomelanocortin (POMC), melanocortin-4 receptor (MC4R), and prohormone convertase, which all alter appetite regulation [115,121]. However, genetic defects directly leading to obesity are rare, representing less than 1% of cases. So, genetic factors play a key secondary role in the development of childhood obesity by increasing an individual’s predisposition to body weight gain in the presence of other factors such as the environmental and behavior [115].

## 8. Fructose and Childhood Obesity

**Figure 3:**
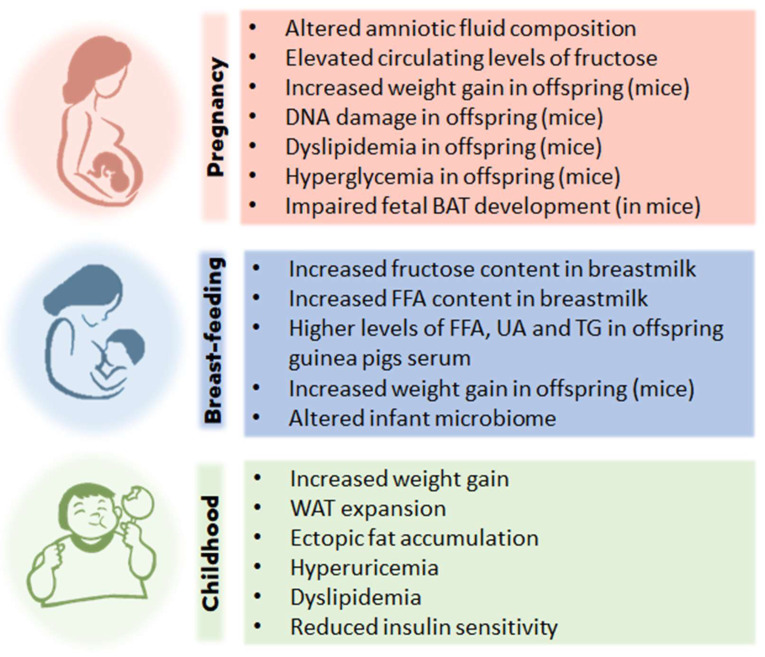
Effects of high fructose intake and childhood obesity. Studies with humans and other animal models support the hypothesis that at different stages of life, from the fetal stage to childhood, excessive consumption of fructose can cause effects that will lead to overweightness and/or childhood obesity. BAT, brown adipose tissue; FFA, free fatty acids; UA, uric acid; TG, triglyceride; WAT, white adipose tissue.

High fructose ingestion and childhood obesity are linked via several paths taken at different stages of a child’s life (Figure 3), even before birth, since fructose crosses the placental barrier [122] and fetal membranes exhibit nutrient transporter expression profiles like the placenta [123]. Supplementation of the maternal diet with carbohydrates (glucose, fructose, or both) provoked a significant increase in amniotic fluid glucose and a significant decrease in amniotic fluid uric acid as the level of carbohydrates increased in the maternal diet [124]. Furthermore, in this study, the glucose content of amniotic fluid was predictive of fetal body weight [124]. Thus, maternal excessive intake of fructose may affect fetal development very early, impacting the developmental patterning of fetal adipose tissue with its highly adipogenic effect [70]. Whether this intrauterine exposure to excess fructose modulates the expression of adipogenic factors such as C/EBPα, C/EBPβ, C/EBPδ, KLF5, KLF9, PPARγ and Zfp423 in fetal adipose tissue remains a gap in knowledge.

The impact of fructose consumption during pregnancy and the impact on fetal metabolic programming is of particular concern. Animal studies have shown that the offspring of mice fed fructose (via fructose syrup) during pregnancy and lactation consumed more food and gained more weight than controls [125,126]. Furthermore, offspring from the mice fed a high-fructose diet showed evidence of DNA damage in blood, liver, kidney, and brain [125] or induced dyslipidemia and hyperglycemia [126], all suggestive of increased long-term susceptibility to cardiometabolic disease. High fructose consumption during pregnancy and lactation was also associated with hypertension in offspring during adulthood, thought to be due to fetal renal programming [127]. Additionally, in pregnant mice, consumption of water with 20% of added fructose resulted in elevated circulating levels of fructose in dams and their fetuses, which led to impaired fetal BAT development, attenuated diet-induced thermogenesis, and metabolic disorders in adult offspring [128]. 

Although the evidence in humans is less clear, a Norwegian study [129] demonstrated an increased risk of preterm birth when pregnant women regularly consumed SSB (high in sucrose/fructose). It is unclear whether the risks were associated directly with the beverages or indirectly due to other dietary or socioeconomic factors, as the regular intake of SSBs is associated with inferior quality diets and poorer socioeconomic status [128]. In China, a significantly increased risk of gestational diabetes was found in pregnant women with higher fasting serum fructose; however, the authors believe that the higher serum fructose was due to the endogenous production of fructose rather than an association with diet [130]. 

An American study [131] assessed the intake of SSBs and fructose in 1068 pregnant women using a food frequency questionnaire. Higher consumption of SSBs was associated with non-white ethnicity, younger maternal age, lower education, and income, plus higher pre-pregnancy BMI. Furthermore, they found that higher intake of SSBs (odds ratio, 1.70; 95% confidence interval, 1.08–2.67) and total fructose (odds ratio, 1.58; 95% confidence interval, 0.98–2.53) during pregnancy was associated with an increased prevalence of asthma in offspring during mid-childhood [131]. Cohen et al. [132] found that sugar consumption during pregnancy, especially from SSBs, adversely affected childhood cognition scores in offspring. 

In terms of food, breast milk can be the child’s first contact with fructose if the mother consumes foods excessively rich in this nutrient, with negative consequences for weight control in progeny. Although the presence of fructose in breast milk does not eliminate the many other benefits of exclusive breastfeeding, the possibility of this nutrient interfering with a child’s taste formation cannot be ruled out since the foods eaten by the mother during pregnancy and lactation form the basis of the child’s weaning patterns [133]. 

Fructose consumption during lactation may also be problematic, as research [134] has found that the consumption of SSBs during lactation increases the fructose content of breastmilk, and this increase remains for up to five hours post-consumption. So, infants who are breastfed by mothers consuming SSBs will be consuming significantly higher levels of fructose during their early life. In guinea pigs fed a high-fructose diet, an increased FFA content in milk was found, which resulted in offspring with altered serum FFA, as well as increased levels of UA and TG [135]. Goran et al. [136] found higher growth in infants at 6 months of age (an increase in both lean and fat mass) when fructose was identified in breastmilk. Moreover, high consumption of SSBs in women during early lactation was associated with lower neurological development scores in infants at 24 months of age [111].

After birth, the development of an infant’s gut microbiota becomes a very important issue. When compared with human milk or traditional lactose-based infant formula, consumption of lactose-reduced infant formula with added corn syrup for a period of six months was found to shape an infant’s microbiome prematurely and was directly associated with the consumption of a high-fat and high-carbohydrate diet during childhood [137]. Alternatively, the numerous human milk oligosaccharides serve as substrate for the proliferation of beneficial bacteria, contributing to an intestinal microbiota composition with health benefits for the breastfed neonate [138]. Considering that fructose is not the most abundant sugar found in human milk (lactose is made of glucose and galactose), the early introduction of fructose into an infant’s diet certainly constitutes a risk factor for childhood obesity.

Moreover, consumers erroneously tend to perceive fructose as a “natural” nutrient [139] originating from fruits and therefore consider it neutral or even beneficial to health [140]. Conversely, SSB intake was correlated with a healthy diet and physical activity among adolescents [141], while a study of 548 children over 19 months showed an association between SSB consumption and obesity, increasing BMI by 0.24 kg/m^2^ (*p* = 0.03) with a 60% rise in obesity (*p* = 0.02) [142]. This evidence indicates the need for reducing fructose intake, already in the early stages of life, to limit the development of childhood obesity.

## 9. What Can We Do to Reduce Fructose Consumption?

Studies have shown that a reduction in fructose and/or free sugar intake might contribute to diminishing metabolic disorders and obesity both in adults and children [54,143]. In that matter, a possible recommendation is consuming foods and beverages sweetened with low- and no-calorie sweeteners (LNCS) such as aspartame, saccharin, and sucralose. The use of these alternative sweeteners limits the consumption of simple carbohydrates and energy intake, improving blood glucose, body weight, and cardiometabolic status [144,145]. However, studies with children are still very scarce, so there is no specific consumption recommendation for this age group [146].

However, the use of LNCS as sweeteners does not represent a complete problem-solving approach. Both caloric and non-caloric sweeteners used in dairy products consumed by Mexican children were associated with the development of obesity and other metabolic disorders [147]. This same study found that the non-caloric sweetener sucralose tends to reduce weight gain but does not alter glycemia, while the caloric sweetener polydextrose exhibited antioxidant, antihypertensive, and antidiabetic properties [147]. In contrast, in a systematic review, Sievenpiper and colleagues did not find clear evidence to justify replacing fructose with glucose in the diet, given the different metabolic effects between them [148]. Considering the variable effects between all types of sweeteners, caloric or non-caloric, and that the general population is unaware of their characteristics and metabolic effects [147], the implementation of educational strategies is fundamental to offer reliable scientific information that will guide better food and beverage choices by parents and children.

Several countries, including the UK, in 2018, have introduced financial penalties (a sugar levy) to dissuade the food industry from adding free sugar to food and beverages, aiming to reduce free sugar consumption by 20% [54]. Mexico also introduced a similar system in 2014, adding a 15% cost to SSBs to discourage consumer purchase [149]. Both countries have observed a reduction in sugar intake since the introduction of the levy, by 4–12% in Mexico [149] and 10% in the UK [54]. In accordance, a meta-analysis also concluded that the implementation of taxes in SSBs around the world is an effective approach for reducing SSB purchase and intake [150].

A Canadian study with 1000 adolescents and young adults (aged 16–30) concluded that consumers appear to base healthiness perceptions on a sweetener’s level of “naturalness” rather than energy content, probably due to their perception of the level of product processing. Most of the respondents perceived HFCS (63.9%) and aspartame (52.4%) as less healthy than table sugar, and the perception of “naturalness” has important implications for understanding consumer preferences [151]. Therefore, an effective approach for limiting SSB consumption among young people might involve warning labels that include calorie information [152,153]. A study conducted in six countries (Australia, Canada, Chile, Mexico, the UK, and the United States) with 10,762 children aged 10–17 showed that different types of front-of-package labels induced the participants to perceive the product as unhealthy, especially with “high in” labels with intuitive symbols [152]. Additionally, another study performed with 2002 parents of children aged 11–16 in the UK showed that SSB selection by parents for their children was lower when labels contained an image-based warning (35%) compared with no label (49%) or calorie information (43.5%) [153]. 

Considering the different strategies, a systematic review evaluated types of sugar reduction initiatives from 22 countries in the Eastern Mediterranean Region and, from this total, 21 countries (95%) implemented at least one type of initiative [153]. Among those initiatives, campaigns for consumer education and awareness, led mainly by governmental entities, were the most common (71%). Additional strategies involved sugar subsidies’ elimination (67%), taxation (62%), food product reformulation, marketing regulation, and food labeling [154]. In fact, many approaches might be helpful for reducing fructose and/or free sugar consumption if they consider the socioeconomic characteristics and food culture of the population. These findings point to the importance of guidance from parents and tutors regarding healthy consumption of SSB, but also the requirement that the population, in general, have access to adequate information about food, its components, and the consequences of its ingestion for people’s health.

## 10. Conclusions

High fructose intake has several different harmful effects on metabolism and adipose tissue function, including the stimulation of adipogenesis and lipogenesis that ultimately can lead to WAT accumulation and obesity. Early exposition to high fructose levels from the intrauterine environment and breastfeeding (both associated with maternal fructose consumption) and in childhood all represent a significant concern for childhood obesity development and its current prevalence globally. In this review, we explored the multiple relationships between excessive fructose ingestion and childhood obesity with particular interest in WAT pathophysiology. Finally, we looked at common strategies used in different countries aiming to reduce fructose consumption and to help prevent childhood obesity. Therefore, we highlight the importance of ensuring that the population has sufficient access to reliable information about the food they are consuming, including nutrient content indicated on food labels. Allied with governmental initiatives such as sugar levies, consumers also need to have reliable knowledge about diet via scientific education to be able to make good choices about what foods should or should not enter their homes. A population able to choose high-quality food will be a population capable of preventing and managing childhood obesity.

## Data Availability

No new data were created or analyzed in this manuscript. Data sharing is not applicable to this review article.

## References

[1] World Obesity Federation World Obesity Atlas 2023. https://data.worldobesity.org/publications/?cat=19.

[2] Apperley L.J., Blackburn J., Erlandson-Parry K., Gait L., Laing P., Senniappan S. (2022). Childhood obesity: A review of current and future management options. Clin. Endocrinol..

[3] Thomas-Eapen N. (2021). Childhood Obesity. Prim. Care-Clin. Off. Pract..

[4] Pandita A., Sharma D., Pandita D., Pawar S., Tariq M., Kaul A. (2016). Childhood obesity: Prevention is better than cure. Diabetes Metab. Syndr. Obes..

[5] Sahoo K., Sahoo B., Choudhury A., Sofi N., Kumar R., Bhadoria A. (2015). Childhood obesity: Causes and consequences. J. Fam. Med. Prim. Care.

[6] Blüher M. (2019). Obesity: Global epidemiology and pathogenesis. Nat. Rev. Endocrinol..

[7] Lin X., Li H. (2021). Obesity: Epidemiology, Pathophysiology, and Therapeutics. Front. Endocrinol..

[8] Johnson R.J., Segal M.S., Sautin Y., Nakagawa T., Feig D.I., Kang D.H., Gersch M.S., Benner S., Sánchez-Lozada L.G. (2007). Potential role of sugar (fructose) in the epidemic of hypertension, obesity and the metabolic syndrome, diabetes, kidney disease, and cardiovascular disease1-3. Am. J. Clin. Nutr..

[9] Pereira R.M., Botezelli J.D., da Cruz Rodrigues K.C., Mekary R.A., Cintra D.E., Pauli J.R., Da Silva A.S.R., Ropelle E.R., De Moura L.P. (2017). Fructose consumption in the development of obesity and the effects of different protocols of physical exercise on the hepatic metabolism. Nutrients.

[10] Helsley R.N., Moreau F., Gupta M.K., Radulescu A., Debosch B., Softic S. (2020). Tissue-Specific Fructose Metabolism in Obesity and Diabetes. Curr. Diabetes Rep..

[11] Czerwonogrodzka-Senczyna A., Rumińska M., Majcher A., Credo D., Jeznach-Steinhagen A., Pyrżak B. (2019). Fructose Consumption and Lipid Metabolism in Obese Children and Adolescents. Adv. Exp. Med. Biol..

[12] Johnson R.J., Sánchez-lozada L.G., Andrews P., Lanaspa M.A. (2017). Perspective: A Historical and Scientific Perspective of Sugar and Its Relation with Obesity and Diabetes. Adv. Nutr..

[13] Kwok K.H., Lam K.S., Xu A. (2016). Heterogeneity of white adipose tissue: Molecular basis and clinical implications. Exp. Mol. Med..

[14] Schoettl T., Fischer I.P., Ussar S. (2018). Heterogeneity of adipose tissue in development and metabolic function. J. Exp. Biol..

[15] Cannon B., Nedergaard J. (2004). Brown adipose tissue: Function and physiological significance. Physiol. Rev..

[16] Ikeda K., Maretich P., Kajimura S. (2018). The Common and Distinct Features of Brown and Beige Adipocytes. Trends Endocrinol. Metabol..

[17] Clemente-Suárez V.J., Redondo-Flórez L., Beltrán-Velasco A.I., Martín-Rodríguez A., Martínez-Guardado I., Navarro-Jiménez E., Laborde-Cárdenas C.C., Tornero-Aguilera J.F. (2023). The role of adipokines in health and disease. Biomedicines.

[18] Song Z., Xiaoli A.M., Yang F. (2018). Regulation and metabolic significance of De Novo lipogenesis in adipose tissues. Nutrients.

[19] Poissonnet C.M., Burdi A.R., Garn S.M. (1984). The chronology of adipose tissue appearance and distribution in the human fetus. Early Hum. Dev..

[20] Billon N., Dani C. (2012). Developmental origins of the adipocyte lineage: New insights from genetics and genomics studies. Stem Cell Rev. Rep..

[21] Ghaben A.L., Scherer P.E. (2019). Adipogenesis and metabolic health. Nat. Rev. Mol. Cell. Biol..

[22] Cristancho A.G., Lazar M.A. (2011). Forming functional fat: A growing understanding of adipocyte differentiation. Nat. Rev. Mol. Cell. Biol..

[23] Knittle J.L., Timmers K., Ginsberg-Fellner F., Brown R.E., Katz D.P. (1979). The growth of adipose tissue in children and adolescents. Cross-sectional and longitudinal studies of adipose cell number and size. J. Clin. Investig..

[24] Cameron M., Demerath E.W. (2002). Critical Periods in Human Growth and Their Relationship to Diseases of Aging. Am. J. Phys. Anthropol..

[25] Spalding K.L., Arner E., Westermark P.O., Bernard S., Buchholz B.A., Bergmann O., Blomqvist L., Hoffstedt J., Näslund E., Britton T. (2008). Dynamics of fat cell turnover in humans. Nature.

[26] Jeffery E., Church C.D., Holtrup B., Colman L., Rodeheffer M.S. (2015). Rapid depot-specific activation of adipocyte precursor cells at the onset of obesity. Nat. Cell Biol..

[27] Schwenk R.W., Holloway G.P., Luiken J.J.F.P., Bonen A., Glatz J.F.C. (2010). Fatty acid transport across the cell membrane: Regulation by fatty acid transporters. Prostaglandins Leukot. Essent. Fat. Acids.

[28] Ameer F., Scandiuzzi L., Hasnain S., Kalbacher H., Zaidi N. (2014). De novo lipogenesis in health and disease. Metabolism.

[29] Bódis K., Roden M. (2018). Energy metabolism of white adipose tissue and insulin resistance in humans. Eur. J. Clin. Investig..

[30] Hirsch J., Farquhar J.W., Ahrens Jr E.H., Peterson M.L., Stoffel W. (1960). Studies of adipose tissue in man. A microtechnic for sampling and analysis. Am. J. Clin. Nutr..

[31] Schaefer-Graf U.M., Meitzner K., Ortega-Senovilla H., Graf K., Vetter K., Abou-Dakn M., Herrera E. (2011). Differences in the implications of maternal lipids on fetal metabolism and growth between gestacional diabetes mellitus and control pregnancies. Diabet. Med..

[32] Fruhbeck G., Mendez-Gimenez L., Fernandez-Formoso J.A., Fernandez S., Rodriguez A. (2014). Regulation of adipocyte lipolysis. Nutr. Res. Rev..

[33] Bolsoni-Lopes A., Alonso-Vale M.I. (2015). Lipolysis and lipases in white adipose tissue—An update. Arch. Endocrinol. Metab..

[34] Desoye G., Herrera E. (2021). Adipose tissue development and lipid metabolism in the human fetus: The 2020 perspective focusing on maternal diabetes and obesity. Prog. Lipid Res..

[35] Marcus C., Ehren H., Bolme P., Arner P. (1988). Regulation of lipolysis during the neonatal period: Importance of thyrotropin. J. Clin. Investig..

[36] Barreiros R.C., Bossolan G., Trindade C.E.P. (2005). Fructose in humans: Metabolic effects, clinical utilization, and associated inherent errors. Rev. Nutr..

[37] Herman M.A., Birnbaum M.J. (2021). Molecular aspects of fructose metabolism and metabolic disease. Cell Metab..

[38] Hallfrisch J. (1990). Metabolic effects of dietary fructose. FASEB J..

[39] Mueckler M. (1994). Facilitative glucose transporters. Eur. J. Biochem..

[40] Mueckler M., Thorens B. (2013). Molecular Aspects of Medicine The SLC2 (GLUT) family of membrane transporters. Mol. Asp. Med..

[41] Truswell S., Thorburn A.W. (1988). Incomplete absorption of pure fructose in healthy subjects. Am. J. Clin. Nutr..

[42] Ferraris R.P., Choe J., Patel C.R. (2018). Intestinal Absorption of Fructose. Annu. Rev. Nutr..

[43] Hannou S.A., Mckeown N.M., Herman M.A., Hannou S.A., Haslam D.E., Mckeown N.M., Herman M.A. (2018). Fructose metabolism and metabolic disease. J. Clin. Investig..

[44] Diggle C.P., Shires M., Leitch D., Brooke D., Carr I.M., Markham A.F., Hayward B.E., Asipu A., Bonthron D.T. (2009). Ketohexokinase: Expression and localization of the principal fructose-metabolizing enzyme. J. Histochem. Cytochem..

[45] Jang C., Hui S., Lu W., Cowan A.J., Morscher R.J., Lee G., Liu W., Tesz G.J., Birnbaum M.J., Rabinowitz J.D. (2019). The small intestine converts dietary fructose into glucose and organic acids. Cell Metabol..

[46] Bidwell A.J. (2017). Chronic fructose ingestion as a major health concern: Is a sedentary lifestyle making it worse? A Review. Nutrients.

[47] Duro D., Rising R., Cedillo M., Lifshitz F. (2002). Association between infantile colic and carbohydrate malabsorption from fruit juices in infancy. Pediatrics.

[48] Gaby A.R. (2005). Adverse effects of dietary fructose. Altern. Med. Rev..

[49] Semnani-Azad Z., Khan T.A., Mejia S.B., de Souza R.J., Leiter L.A., Kendall C.W., Hanley A.J., Sievenpiper J.L. (2020). Association of major food sources of fructose-containing sugars with incident metabolic syndrome: A systematic review and meta-analysis. JAMA Netw. Open.

[50] Merino B., Fernández-Díaz C.M., Cózar-Castellano I., Perdomo G. (2019). Intestinal fructose and glucose metabolism in health and disease. Nutrients.

[51] Fidler Mis N., Braegger C., Bronsky J., Campoy C., Domellöf M., Embleton N.D., Hojsak I., Hulst J., Indrio F., Lapillonne A. (2017). Sugar in Infants, Children and Adolescents: A Position Paper of the European Society for Paediatric Gastroenterology, Hepatology and Nutrition Committee on Nutrition. J. Pediatr. Gastroenterol. Nutr..

[52] World Health Organization Sugars Intake for Adults and Children. https://www.who.int/publications/i/item/9789241549028.

[53] UK’s Scientific Advisory Committee on Nutrition Carbohydrates and Health. https://assets.publishing.service.gov.uk/government/uploads/system/uploads/attachment_data/file/445503/SACN_Carbohydrates_and_Health.pdf.

[54] Young J., Scott S., Clark L., Lodge J.K. (2022). Associations between free sugar intake and markers of health in the UK population: An analysis of the National Diet and Nutrition Survey rolling programme. Br. J. Nutr..

[55] Kmietowicz Z. (2012). Countries that use large amounts of high fructose corn syrup have higher rates of type 2 diabetes. BMJ.

[56] Bragança M.L.B.M., Bogea E.G., de Almeida Fonseca Viola P.C., dos Santos Vaz J., Confortin S.C., Menezes A.M.B., Gonçalves H., Bettiol H., Barbieri M.A., Cardoso V.C. (2023). High Consumption of Sugar-Sweetened Beverages Is Associated with Low Bone Mineral Density in Young People: The Brazilian Birth Cohort Consortium. Nutrients.

[57] Taskinen M.R., Packard C.J., Borén J. (2019). Dietary fructose and the metabolic syndrome. Nutrients.

[58] Klein A.V., Kiat H. (2015). The mechanisms underlying fructose-induced hypertension: A review. J. Hypertens..

[59] Ichigo Y., Takeshita A., Hibino M., Nakagawa T., Hayakawa T., Patel D., Field C.J., Shimada M. (2019). High-fructose diet-induced hypertriglyceridemia is associated with enhanced hepatic expression of ACAT2 in Rats. Physiol. Res..

[60] DiStefano J.K. (2020). Fructose-mediated effects on gene expression and epigenetic mechanisms associated with NAFLD pathogenesis. Cell. Mol. Life Sci..

[61] Muriel P., López-sánchez P., Ramos-tovar E. (2021). Fructose and the liver. Int. J. Mol. Sci..

[62] Lê K.A., Ith M., Kreis R., Faeh D., Bortolotti M., Tran C., Boesch C., Tappy L. (2009). Fructose overconsumption causes dyslipidemia and ectopic lipid deposition in healthy subjects with and without a family history of type 2 diabetes. Am. J. Clin. Nutr..

[63] Basciano H., Federico L., Adeli K. (2005). Fructose, insulin resistance, and metabolic dyslipidemia. Nutr. Metab..

[64] Catena C., Giacchetti G., Novello M., Colussi G., Cavarape A., Sechi L.A. (2003). Cellular Mechanisms of Insulin Resistance in Rats with Fructose-Induced Hypertension. Am. J. Hypertens..

[65] Ueno M., Bezerra R.M.N., Silva M.S., Tavares D.Q., Carvalho C.R., Saad M.J.A. (2000). A high-fructose diet induces changes in pp185 phosphorylation in muscle and liver of rats. Braz. J. Med. Biol. Res..

[66] Russo E., Leoncini G., Esposito P., Garibotto G., Pontremoli R., Viazzi F. (2020). Fructose and uric acid: Major mediators of cardiovascular disease risk starting at pediatric age. Int. J. Mol. Sci..

[67] Spiga R., Marini M.A., Mancuso E., Di Fatta C., Fuoco A., Perticone F., Andreozzi F., Mannino G.C., Sesti G. (2017). Uric Acid Is Associated with Inflammatory Biomarkers and Induces Inflammation Via Activating the NF-κB Signaling Pathway in HepG2 Cells. Arter. Thromb. Vasc. Biol..

[68] Wang Y., Qi W., Song G., Pang S., Peng Z., Li Y., Wang P. (2020). High-fructose diet increases inflammatory cytokines and alters gut microbiota composition in rats. Mediat. Inflamm..

[69] Hernández-Díazcouder A., Romero-Nava R., Carbó R., Sánchez-Lozada L.G., Sánchez-Muñoz F. (2019). High fructose intake and adipogenesis. Int. J. Mol. Sci..

[70] Jürgens H., Haass W., Castañeda T.R., Schürmann A., Koebnick C., Dombrowski F., Otto B., Nawrocki A.R., Scherer P.E., Spranger J. (2005). Consuming fructose-sweetened beverages increases body adiposity in mice. Obes. Res..

[71] Pektas M.B., Koca H.B., Sadi G., Akar F. (2016). Dietary Fructose Activates Insulin Signaling and Inflammation in Adipose Tissue: Modulatory Role of Resveratrol. Biomed. Res. Int..

[72] Yahia H., Hassan A., El-Ansary M.R., Al-Shorbagy M.Y., El-Yamany M.F. (2020). IL-6/STAT3 and adipokine modulation using tocilizumab in rats with fructose-induced metabolic syndrome. Naunyn. Schmiedebergs Arch. Pharmacol..

[73] Miranda C.S., Silva-Veiga F.M., Santana-Oliveira D.A., Fernandes-da-Silva A., Brito G.C., Martins F.F., Souza-Mello V. (2023). Chronic Excessive Fructose Intake Maximizes Brown Adipocyte Whitening but Causes Similar White Adipocyte Hypertrophy Than a High-Fat Diet in C57BL/6 Mice. J. Am. Nutr. Assoc..

[74] Santos M.P., Cauduro L.F.R., Ferreira M.M., Martucci L.F., Vecchiatto B., Vilas-boas E.A., Américo A.L.V., Pereira R.O., Rogero M.M., Fiorino P. (2023). Effect of Low-Dose Progesterone on Glycemic Metabolism, Morphology and Function of Adipose Tissue and Pancreatic Islets in Diet-Induced Obese Female Mice. Front. Biosci. (Landmark Ed.).

[75] Crescenzo R., Bianco F., Coppola P., Mazzoli A., Valiante S., Liverini G., Iossa S. (2014). Adipose tissue remodeling in rats exhibiting fructose-induced obesity. Eur. J. Nutr..

[76] Zubiría M.G., Alzamendi A., Moreno G., Rey M.A., Spinedi E., Giovambattista A. (2016). Long-term fructose intake increases adipogenic potential: Evidence of direct effects of fructose on adipocyte precursor cells. Nutrients.

[77] London E., Castonguay T.W. (2011). High fructose diets increase 11β-hydroxysteroid dehydrogenase type 1 in liver and visceral adipose in rats within 24-h exposure. Obesity.

[78] Legeza B., Balázs Z., Odermatt A. (2014). Fructose promotes the differentiation of 3T3-L1 adipocytes and accelerates lipid metabolism. FEBS Lett..

[79] Prince P.D., Santander Y.A., Gerez E.M., Höcht C., Polizio A.H., Mayer M.A., Taira C.A., Fraga C.G., Galleano M., Carranza A. (2017). Fructose increases corticosterone production in association with NADPH metabolism alterations in rat epididymal white adipose tissue. J. Nutr. Biochem..

[80] Lee M.J., Pramyothin P., Karastergiou K., Fried S.K. (2014). Deconstructing the roles of glucocorticoids in adipose tissue biology and the development of central obesity. Biochim. Biophys. Acta-Mol. Basis Dis..

[81] Park Y.-K., Ge K. (2017). Glucocorticoid Receptor Accelerates, but Is Dispensable for, Adipogenesis. Mol. Cell. Biol..

[82] Du L., Heaney A.P. (2012). Regulation of adipose differentiation by fructose and GluT5. Mol. Endocrinol..

[83] Meneses M.J., Sousa-Lima I., Jarak I., Raposo J.F., Alves M.G., Macedo M.P. (2022). Distinct impacts of fat and fructose on the liver, muscle, and adipose tissue metabolome: An integrated view. Front. Endocrinol..

[84] Li J.X., Ke D.Z., Yao L., Wang S., Ma P., Liu L., Zuo G.W., Jiang L.R., Wang J.W. (2017). Response of genes involved in lipid metabolism in rat epididymal white adipose tissue to different fasting conditions after long-term fructose consumption. Biochem. Biophys. Res. Commun..

[85] Mazzoli A., Di Porzio A., Gatto C., Crescenzo R., Nazzaro M., Spagnuolo M.S., Baccigalupi L., Ricca E., Amoresano A., Fontanarosa C. (2023). Skeletal muscle insulin resistance and adipose tissue hypertrophy persist beyond the reshaping of gut microbiota in young rats fed a fructose-rich diet. J. Nutr. Biochem..

[86] Kovačević S., Brkljačić J., Vojnović Milutinović D., Gligorovska L., Bursać B., Elaković I., Djordjevic A. (2021). Fructose Induces Visceral Adipose Tissue Inflammation and Insulin Resistance Even without Development of Obesity in Adult Female but Not in Male Rats. Front. Nutr..

[87] Baldwin W., McRae S., Marek G., Wymer D., Pannu V., Baylis C., Johnson R.J., Sautin Y.Y. (2011). Hyperuricemia as a mediator of the proinflammatory endocrine imbalance in the adipose tissue in a murine model of the metabolic syndrome. Diabetes.

[88] Singh S., Sharma A., Guru B., Ahmad S., Gulzar F., Kumar P., Ahmad I., Tamrakar A.K. (2022). Fructose-mediated NLRP3 activation induces inflammation and lipogenesis in adipose tissue. J. Nutr. Biochem..

[89] Kuzma J.N., Cromer G., Hagman D.K., Breymeyer K.L., Roth C.L., Foster-Schubert K.E., Holte S.E., Weigle D.S., Kratz M. (2016). No differential effect of beverages sweetened with fructose, high-fructose corn syrup, or glucose on systemic or adipose tissue inflammation in normal-weight to obese adults: A randomized controlled trial. Am. J. Clin. Nutr..

[90] Manna P., Jain S.K. (2015). Obesity, Oxidative Stress, Adipose Tissue Dysfunction, and the Associated Health Risks: Causes and Therapeutic Strategies. Metab. Syndr. Relat. Disord..

[91] Bratoeva K., Radanova M., Merdzhanova A., Donev I. (2017). Protective role of S-Adenosylmethionine against fructose-induced oxidative damage in obesity. J. Mind Med. Sci..

[92] Araoye E., Ckless K. (2016). Effects of High Fructose/Glucose on Nlrp3/Il1β Inflammatory Pathway. J. Young Investig..

[93] Gherghina M.E., Peride I., Tiglis M., Neagu T.P., Niculae A., Checherita I.A. (2022). Uric Acid and Oxidative Stress—Relationship with Cardiovascular, Metabolic, and Renal Impairment. Int. J. Mol. Sci..

[94] Bjelaković G., Beninati S., Pavlović D., Kocić G., Jevtović T., Kamenov, Šaranac L.J., Bjelaković B., Stojanović I., Bašić J. (2007). Glucocorticoids and Oxidative Stress. J. Basic Clin. Physiol. Pharmacol..

[95] Imhoff B.R., Hansen J.M. (2010). Extracellular redox environments regulate adipocyte differentiation. Differentiation.

[96] Han J., Choi H.Y., Dayem A.A., Kim K., Yang G., Won J., Do S.H., Kim J.H., Jeong K.S., Cho S.G. (2017). Regulation of Adipogenesis Through Differential Modulation of ROS and Kinase Signaling Pathways by 3,4′-Dihydroxyflavone Treatment. J. Cell. Biochem..

[97] Zorena K., Jachimowicz-Duda O., Ślęzak D., Robakowska M., Mrugacz M. (2020). Adipokines and obesity. Potential link to metabolic disorders and chronic complications. Int. J. Mol. Sci..

[98] Taylor E.B. (2021). The complex role of adipokines in obesity, inflammation, and autoimmunity. Clin. Sci..

[99] Maslov L.N., Naryzhnaya N.V., Boshchenko A.A., Popov S.V., Ivanov V.V., Oeltgen P.R. (2019). Is oxidative stress of adipocytes a cause or a consequence of the metabolic syndrome?. J. Clin. Transl. Endocrinol..

[100] Rodrigues D.F., do Carmo Henriques M.C., Oliveira M.C., Menezes-Garcia Z., Marques P.E., da Glória Souza D., Menezes G.B., Teixeira M.M., Ferreira A.V.M. (2014). Acute intake of a high-fructose diet alters the balance of adipokine concentrations and induces neutrophil influx in the liver. J. Nutr. Biochem..

[101] Chait A., den Hartigh L.J. (2020). Adipose Tissue Distribution, Inflammation and Its Metabolic Consequences, Including Diabetes and Cardiovascular Disease. Front. Cardiovasc. Med..

[102] Mendoza-herrera K., Florio A.A., Moore M., Marrero A., Tamez M., Bhupathiraju S.N., Mattei J. (2021). The Leptin System and Diet: A Mini Review of the Current Evidence. Front. Endocrinol..

[103] Jian-mei L.I., Chuang W., Qing-hua H.U., Ling-dong K. (2008). Fructose Induced Leptin Dysfunction and Improvement by Quercetin and Rutin in Rats. Chin. J. Nat. Med..

[104] Shapiro A., Tu N., Gao Y., Cheng K., Scarpace P.J. (2011). Prevention and reversal of diet-induced leptin resistance with a sugar-free diet despite high fat content. Br. J. Nutr..

[105] Shapiro A., Mu W., Roncal C., Cheng K., Johnson R.J., Scarpace P.J. (2008). Fructose-induced leptin resistance exacerbates weight gain in response to subsequent high-fat feeding. Am. J. Physiol.-Regul. Integr. Comp. Physiol..

[106] Äijälä M., Malo E., Ukkola O., Bloigu R., Lehenkari P., Autio-Harmainen H., Santaniemi M., Kesäniemi Y.A. (2013). Long-term fructose feeding changes the expression of leptin receptors and autophagy genes in the adipose tissue and liver of male rats: A possible link to elevated triglycerides. Genes Nutr..

[107] Haring S.J., Harris R.B.S. (2011). The relation between dietary fructose, dietary fat and leptin responsiveness in rats. Physiol. Behav..

[108] Miranda C.S., Silva-Veiga F., Martins F.F., Rachid T.L., Mandarim-De-Lacerda C.A., Souza-Mello V. (2020). PPAR-α activation counters brown adipose tissue whitening: A comparative study between high-fat– and high-fructose–fed mice. Nutrition.

[109] Machado T.Q., Pereira-Silva D.C., Goncalves L.F., Fernandes-Santos C. (2019). Brown Adipose Tissue Remodeling Precedes Cardiometabolic Abnormalities Independent of Overweight in Fructose-Fed Mice. Integr. Diabetes Cardiovasc. Dis..

[110] Richard G., Blondin D.P., Syed S.A., Rossi L., Fontes M.E., Fortin M., Phoenix S., Frisch F., Dubreuil S., Guérin B. (2022). High-fructose feeding suppresses cold-stimulated brown adipose tissue glucose uptake independently of changes in thermogenesis and the gut microbiome. Cell Rep. Med..

[111] Berger P.K., Plows J.F., Jones R.B., Alderete T.L., Rios C., Pickering T.A., Fields D.A., Bode L., Peterson B.S., Goran M.I. (2020). Associations of maternal fructose and sugar-sweetened beverage and juice intake during lactation with infant neurodevelopmental outcomes at 24 months. Am. J. Clin. Nutr..

[112] Larqué E., Labayen I., Flodmark C.E., Lissau I., Czernin S., Moreno L.A., Pietrobelli A., Widhalm K. (2019). From conception to infancy—Early risk factors for childhood obesity. Nat. Rev. Endocrinol..

[113] Shaban Mohamed M.A., AbouKhatwa M.M., Saifullah A.A., Hareez Syahmi M., Mosaad M., Elrggal M.E., Dehele I.S., Elnaem M.H. (2022). Risk Factors, Clinical Consequences, Prevention, and Treatment of Childhood Obesity. Children.

[114] Drozdz D., Alvarez-Pitti J., Wójcik M., Borghi C., Gabbianelli R., Mazur A., Herceg-čavrak V., Lopez-Valcarcel B.G., Brzeziński M., Lurbe E. (2021). Obesity and cardiometabolic risk factors: From childhood to adulthood. Nutrients.

[115] Avelar Rodriguez D., Toro Monjaraz E.M., Ignorosa Arellano K.R., Ramirez Mayans J. (2018). Childhood obesity in Mexico: Social determinants of health and other risk factors. BMJ Case Rep..

[116] Lee E.Y., Yoon K.H. (2018). Epidemic obesity in children and adolescents: Risk factors and prevention. Front. Med..

[117] Williams C.B., MacKenzie K.C., Gahagan S. (2014). The effect of maternal obesity on the offspring. Clin. Obs. Gynecol..

[118] Lakshman R., Elks C.E., Ong K.K. (2012). Childhood obesity. Circulation.

[119] Mahumud R.A., Sahle B.W., Owusu-Addo E., Chen W., Morton R.L., Renzaho A.M. (2021). Association of dietary intake, physical activity, and sedentary behaviours with overweight and obesity among 282,213 adolescents in 89 low and middle income to high-income countries. Int. J. Obes..

[120] Mittal M., Jain V. (2021). Management of Obesity and Its Complications in Children and Adolescents. Indian J. Pediatr..

[121] Hemmingsson E. (2018). Early Childhood Obesity Risk Factors: Socioeconomic Adversity, Family Dysfunction, Offspring Distress, and Junk Food Self-Medication. Curr. Obes. Rep..

[122] Kostovski M., Tasic V., Laban N., Polenakovic M., Danilovski D., Gucev Z. (2017). Obesity in childhood and adolescence, genetic factors. Priloz.

[123] Holmberg N.G., Kaplan B., Karvonen M.J., Lind J., Malm M. (1956). Permeability of Human Placenta to Glucose, Fructose, and Xylose. Acta Physiol. Scand..

[124] Lintao R.C.V., Kammala A.K., Vora N., Yaklic J.L., Menon R. (2023). Fetal membranes exhibit similar nutrient transporter expression profiles to the placenta. Placenta.

[125] Magenis M.L., Damiani A.P., de Bem Silveira G., Dagostin L.S., de Marcos P.S., de Souza E., de Roch Casagrande L., Longaretti L.M., Silveira P.C., de Andrade V.M. (2022). Metabolic programming in offspring of mice fed fructose during pregnancy and lactation. J. Dev. Orig. Health Dis..

[126] Koo S., Kim M., Cho H.M., Kim I. (2021). Maternal high-fructose intake during pregnancy and lactation induces metabolic syndrome in adult offspring. Nutr. Res. Pract..

[127] Jia G., Hill M.A., Sowers J.R. (2019). Maternal exposure to high fructose and offspring health. Hypertension.

[128] Wang P., Wu T., Fu Q., Liao Q., Li Y., Huang T., Li Y., Zhou L., Song Z. (2022). Maternal High-Fructose Intake Activates Myogenic Program in Fetal Brown Fat and Predisposes Offspring to Diet-Induced Metabolic Dysfunctions in Adulthood. Front. Nutr..

[129] Englund-Ögge L., Brantsæter A.L., Haugen M., Sengpiel V., Khatibi A., Myhre R., Myking S., Meltzer H.M., Kacerovsky M., Nilsen R.M. (2012). Association between intake of artificially sweetened and sugar-sweetened beverages and preterm delivery: A large prospective cohort study. Am. J. Clin. Nutr..

[130] Zhang H., Li X., Niu Y., Yang Z., Lu Y., Su Q., Qin L. (2022). Fasting serum fructose is associated with risk of gestational diabetes mellitus. BMC Pregnancy Childbirth.

[131] Wright L.S., Rifas-Shiman S.L., Oken E., Litonjua A.A., Gold D.R. (2018). Prenatal and Early Life Fructose, Fructose-Containing Beverages, and Mid childhood Asthma. Ann. Am. Thorac. Soc..

[132] Cohen J.F.W., Rifas-Shiman S.L., Young J., Oken E. (2018). Associations of prenatal and child sugar intake with child cognition. Am. J. Prev. Med..

[133] Koski K.G., Fergusson M.A. (1992). Amniotic fluid composition responds to changes in maternal dietary carbohydrate and is related to metabolic status in term fetal rats. J. Nutr..

[134] Berger P.K., Fields D.A., Demerath E.W., Fujiwara H., Goran M.I. (2018). High-fructose corn syrup-sweetened beverage intake increases 5-hour breast milk fructose concentrations in lactating women. Nutrients.

[135] Smith E.V.L., Dyson R.M., Berry M.J., Gray C. (2020). Fructose Consumption During Pregnancy Influences Milk Lipid Composition and Offspring Lipid Profiles in Guinea Pigs. Front. Endocrinol..

[136] Goran M.I., Martin A.A., Alderete T.L., Fujiwara H., Fields D.A. (2017). Fructose in Breast Milk Is Positively Associated with Infant Body Composition at 6 Months of Age. Nutrients.

[137] Jones R.B., Berger P.K., Plows J.F., Alderete T.L., Millstein J., Fogel J., Iablokov S.N., Rodionov D.A., Osterman A.L., Bode L. (2020). Lactose-reduced infant formula with added corn syrup solids is associated with a distinct gut microbiota in Hispanic infants. Gut Microbes.

[138] Bode L. (2012). Human milk oligosaccharides: Every baby needs a sugar mama. Glycobiology.

[139] Giussani M., Lieti G., Orlando A., Parati G., Genovesi S. (2022). Fructose Intake, Hypertension and Cardiometabolic Risk Factors in Children and Adolescents: From Pathophysiology to Clinical Aspects. A Narrative Review. Front. Med..

[140] Febbraio M.A., Karin M. (2021). “Sweet death”: Fructose as a metabolic toxin that targets the gut-liver axis. Cell Metab..

[141] Ranjit N., Evans M.H., Byrd-Williams C., Evans A.E., Hoelscher D.M. (2010). Dietary and activity correlates of sugar-sweetened beverage consumption among adolescents. Pediatrics.

[142] Berkey C.S., Rockett H.R.H., Field A.E., Gillman M.W., Colditz G.A. (2004). Sugar-added beverages and adolescent weight change. Obes. Res..

[143] Maier I.B., Stricker L., Özel Y., Wagnerberger S., Bischoff S.C., Bergheim I. (2011). A low fructose diet in the treatment of pediatric obesity: A pilot study. Pediatr. Int..

[144] Warshaw H., Edelman S.V. (2021). Practical strategies to help reduce added sugars consumption to support glycemic and weight management goals. Clin. Diabetes.

[145] Rogers P.J., Appleton K.M. (2021). The effects of low-calorie sweeteners on energy intake and body weight: A systematic review and meta-analyses of sustained intervention studies. Int. J. Obes..

[146] World Health Organization (2023). Use of Non-Sugar Sweeteners: WHO Guideline.

[147] Briones-Avila L.S., Moranchel-Hernández M.A., Moreno-Riolobos D., Silva Pereira T.S., Ortega Regules A.E., López K.V., Islas Romero L.M. (2021). Analysis of caloric and noncaloric sweeteners present in dairy products aimed at the school market and their possible effects on health. Nutrients.

[148] Sievenpiper J.L., De Souza R.J., Cozma A.I., Chiavaroli L., Ha V., Mirrahimi A. (2014). Fructose vs. glucose and metabolism: Do the metabolic differences matter?. Curr. Opin. Lipidol..

[149] Colchero M.A., Guerrero-Lopez C.M., Molina M., Rivera J.A. (2016). Beverages sales in Mexico before and after Implementation of a sugar sweetened beverage tax. PLoS ONE.

[150] Teng A.M., Jones A.C., Mizdrak A., Signal L., Genç M., Wilson N. (2019). Impact of sugar-sweetened beverage taxes on purchases and dietary intake: Systematic review and meta-analysis. Obes. Rev..

[151] Goodman S., Vanderlee L., Jones A., White C., Hammond D. (2021). Perceived healthiness of sweeteners among young adults in Canada. Can. J. Diet Pract. Res..

[152] Hock K., Acton R.B., Jáuregui A., Vanderlee L., White C.M., Hammond D. (2021). Experimental study of front-of-package nutrition labels’ efficacy on perceived healthfulness of sugar-sweetened beverages among youth in six countries. Prev. Med. Rep..

[153] Mantzari E., Vasiljevic M., Turney I., Pilling M., Marteau T. (2018). Impact of warning labels on sugar-sweetened beverages on parental selection: An online experimental study. Prev. Med. Rep..

[154] Al-Jawaldeh A., Taktouk M., Naalbandian S., Aguenaou H., Al Hamad N., Almamary S., Al-Tamimi H.A., Alyafei S.A., Barham R., Hoteit M. (2023). Sugar Reduction Initiatives in the Eastern Mediterranean Region: A Systematic Review. Nutrients.

